# Bio-inspired, large scale, highly-scattering films for nanoparticle-alternative white surfaces

**DOI:** 10.1038/srep46637

**Published:** 2017-04-21

**Authors:** Julia Syurik, Radwanul Hasan Siddique, Antje Dollmann, Guillaume Gomard, Marc Schneider, Matthias Worgull, Gabriele Wiegand, Hendrik Hölscher

**Affiliations:** 1 Institute for Microstructure Technology, Karlsruhe Institute of Technology (KIT), Eggenstein-Leopoldshafen, 76344, Germany; 2 Medical Engineering, California Institute of Technology, Pasadena, CA 91125, USA; 3 Light Technology Institute, Karlsruhe Institute of Technology (KIT), Karlsruhe, 76131, Germany; 4 Institute of Catalysis Research and Technology, Karlsruhe Institute of Technology (KIT), Eggenstein-Leopoldshafen, 76344, Germany

## Abstract

Inspired by the white beetle of the genus *Cyphochilus*, we fabricate ultra-thin, porous PMMA films by foaming with CO_2_ saturation. Optimising pore diameter and fraction in terms of broad-band reflectance results in very thin films with exceptional whiteness. Already films with 60 µm-thick scattering layer feature a whiteness with a reflectance of 90%. Even 9 µm thin scattering layers appear white with a reflectance above 57%. The transport mean free path in the artificial films is between 3.5 µm and 4 µm being close to the evolutionary optimised natural prototype. The bio-inspired white films do not lose their whiteness during further shaping, allowing for various applications.

White is the most popular color for today’s industrial products^1^. Being the essential color for interiors, white pigments are widely found in plastics^1^, inks and paints^2^. Additionally, they are frequently used in cosmetics^3^ and even food^4^. In many of these products, their whiteness is achieved by incorporating titanium dioxide (TiO_2_) particles which, due to their high refractive index (RI) of *n* ≈ 2.6, effectively scatter incoming visible light^1^. Fabricating white polymeric films and bulk parts with TiO_2_ particles causes some issues: (i) an extra operational step that increases costs, (ii) complicated disposal procedures due to possible environmental harm by TiO_2_ particles, (iii) pulmonary inflammation effects^5^,^6^, and (iv) suspected health issues^7^. Therefore, particle-free alternatives are of particular interest^8^.

Nature offers a variety of white surfaces, that are built without pigment particles. Cephalopods^9^, insects^10^,^11^ and plants^12^,^13^ utilise structural white color^14^ for camouflage^9^,^15^, communication and mating^11^,^16^, thermoregulation^17^ and pollination promotion^13^. Such natural white colors are caused by scattering layers which consist of highly disordered nanostructures. For example, the beetle *Calothyrza margaritifera* (Westwood, 1848)^18^ has scattering elements with a bead-like shape; the white beetles *Cyphochilus insulanus* (Moser, 1918)^19^ (see Fig. 1a), *Cyphochilus cratacea* (Niijima & Kinoshita, 1923)^20^ and *Lepidiota stigma* (Fabricius, 1798)^21^ have rod-like scatterers; while the petals of some flowers, like *Diphylleia grayi*^12^ and *Laelia purpurata* (Lindl & Paxton)^13^, appear white due to micropores.

In the above mentioned cases, the building materials feature a comparable low refractive index (*n* ≈ 1.55 for chitin^22^ and *n* ≈ 1.73 for melanin^23^ at 600 nm). Despite that, the refractive index contrast between both of these natural materials and the surrounding medium like air is high enough to trigger efficient scattering, even from very thin layers. The famous white beetles of the genus *Cyphochilus*, for instance, produce their brilliant white appearance with porous chitin scales, which are only 7 ± 1.5 *μ*m thick^24^ but effect a reflection of 65–70%^21^,^25^. Figure 1 shows the species *Cyphochilus insulanus* and scanning electron microscopy (SEM) images of its scales and the their inner structure.

The fact, that nature can build such thin, yet very efficient scattering layers from low RI materials, suggests that similar effect can be reached within thin layers of other materials with low refractivity. Indeed, various pigment-free scattering surfaces have already been produced from different materials, including polymers: polystyrene microspheres^26^,^27^, silicon oxynitride nanowires^28^, electrospun nanofibers^29^,^30^, nanoporous polyimide^31^ and collapsed micropillars^32^. Proposed applications range from paper coatings^29^,^30^ and Lambertian-like reflectors for light trapping in solar cells^26^ to light diffusers attached to the frontside of light emitting diodes^31^,^33^. However, none of the suggested techniques can produce both thin and flexible white film on large scales.

Here, we present an approach to whiten thin polymer foils via saturation by supercritical carbon dioxide (SC-CO_2_), which is an industrial process suitable for large scale fabrication. It is already a well-established method for producing foams from polymers^34^,^35^ including polystyrene (PS)^36^ and poly(methyl methacrylate) (PMMA)^37^. The morphology of the final foam depends on the polymer itself, its solubility in CO_2_^34^ and the so-called plasticisation effect^38^, that is, the decrease of glass transition temperature of the polymer in CO_2_. Because solubility and plasticisation depend on temperature and pressure, these process parameters allow one to control the size and the density of the pores in the foam. Although homogeneous nanoporous polymers have been already produced in bulk^39^,^40^,^41^, saturating thin layers is a challenge. Since the concentration of CO_2_ in the polymer drops extremely fast during the depressurisation step, it might be not sufficient to form the homogeneous nanofoam needed for optical applications.

We tailored the saturation parameters for the fabrication of thin and white nanofoams made from PMMA because it is a common, often used polymer. The process parameters effectively control size, shape and wall thickness of the pores which act as scattering elements. Although the refractive index of PMMA (*n* = 1.49 at 600 nm)^42^,^43^ is even lower than that of chitin (*n* = 1.55 at 600 nm)^22^ the films are perfectly white even for comparable thin film thicknesses. In order to demonstrate the processability of our porous white films, we fabricated ultra-white microchannels from them.

## Results and Discussion

**Figure 2:**
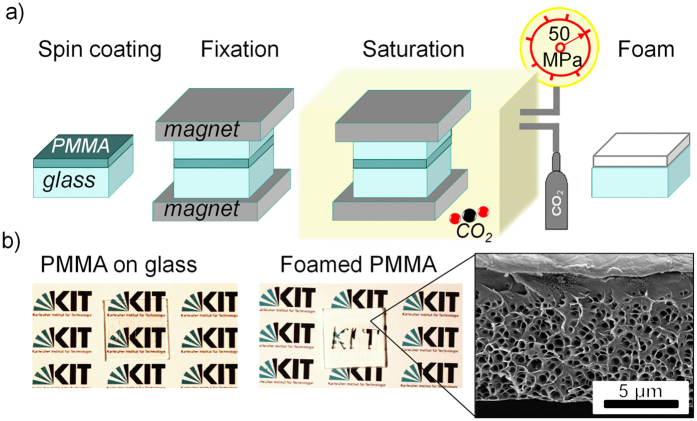
(**a**) Schematic of the foaming process by saturation with CO_2_. PMMA resist spin-coated on glass is covered with the second glass slide and clamped between two neodymium magnets in order to avoid deformations of the films during foaming. Such a stack was placed in a home-built high pressure cell connected to a CO_2_ balloon and a pump. Applying suitable pressure up to 50 MPa, temperature and saturation times nanocellular foam is formed during the final rapid depressurisation step. (**b**) The result of the foaming can be observed by the whitening of the previously transparent PMMA film.

We prepared white PMMA films with the process shown in Fig. 2a. The films, spin-coated on a glass substrate and covered with another glass slide for protection, were fixed between two strong magnets and foamed in three steps^34^,^44^,^45^: (i) saturation of the polymer with CO_2_, (ii) formation of nuclei in the polymer as a result of supersaturation, (iii) pore growth due to diffusion of CO_2_ from PMMA to pores. The produced films change their appearance from transparent to white or whitish (Fig. 2b), induced by light scattering on multiple polymer-air interphases (i.e. on the pores). Therefore, light scattering in foamed PMMA depends on pore morphology. The better the scattering efficiency of the porous film, the whiter it appears to the eye.

### Optimising Pore Morphology through Saturation Pressure

**Figure 3:**
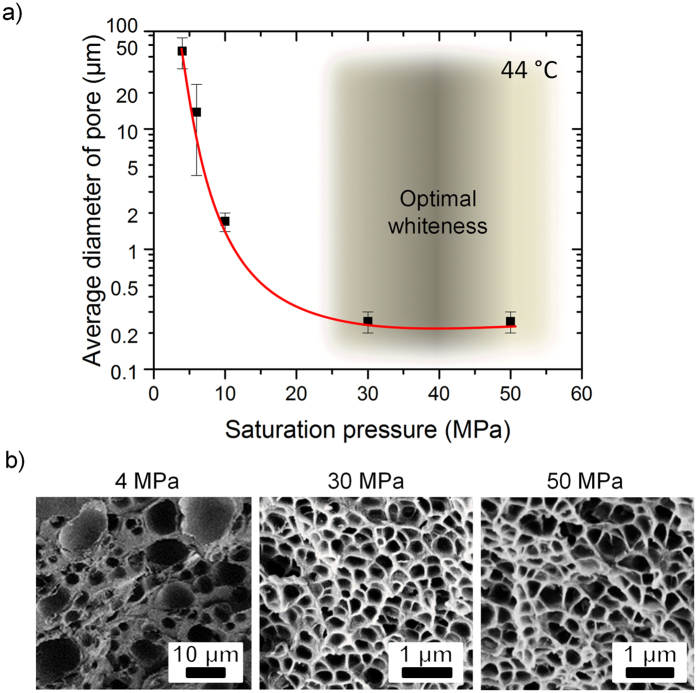
(**a**) Mean pore diameter as a function of saturation pressure. Pressures above 30 MPa result in pore diameters in the nanometer range, which does not decrease for higher pressures. The pressure range giving the whitest films, i.e. dense, homogeneous, sub-nanometer sized pores, is shadowed. (**b**) SEM images of the foamed films for three applied pressures, indicated above the images. We observed an increase of pore homogeneity for high pressures, i.e. above 30 MPa, and insignificant change in morphology for higher pressures.

In a first step, we optimised the saturation pressure because it has stronger influence on the foaming process than the temperature^37^,^46^. For that, we saturated free standing PMMA films with nominal thickness of 100 *μ*m with a pressure ranging from 4 to 50 MPa at 44 °C for 3 h. Figure 3 compares the resulting pore diameters and foam structures. We applied two saturation techniques: pressure-induced (PIPhS) and thermally induced phase separation (TIPhS)^37^, depending on the pressure range.

For both techniques the pore (nuclei) formation starts at the supersaturation condition (*T* > *T*_g_), when the surrounding temperature is higher than the glass transition temperature *T*_g_, and the actual concentration of CO_2_ in the polymer is above its equilibrium value. Thus, the temperature difference Δ*T* = *T* − *T*_g_, yields the energy of the foaming process, where *T*_g_ is a function of pressure. For lower pressures (below 70 MPa) the supersaturation can be created by fast temperature rise (TIPhS), for higher saturation pressures (above 70 MPa) - by a rapid pressure drop (PIPhS). The latter is possible due to the plastification effect of SC-CO_2_ causing a drop of the effective *T*_g_ of PMMA below 40 °C. The pore growth stops when Δ*T* < 0, freezing the resulting nano- or microstructure. In general, PIPhS has the two advantages that no extra heating step is required, as supersaturation occurs during depressurisation and the pore growth stops automatically when the ambient pressure drops and weakens the plastification effect, without additional cooling. We performed PIPhS for the samples saturated above 10 MPa but this is only applicable when SC-CO_2_ is used. Therefore, at lower pressures of 4 to 6 MPa (gaseous CO_2_) we created supersaturation conditions with TIPhS, by heating the sample above the *T*_g_ of PMMA (105 °C) for 10 s directly after the depressurisation step. The foaming process was stopped by cooling the samples in icy water^36^.

The pore diameters of the samples prepared in that way are shown in Fig. 3 together with scanning electron microscopy images of their internal structure. The mean pore diameter drops from 45 ± 13 *μ*m at 4 MPa to 14 ± 10 *μ*m at 6 MPa (Fig. 3a). Broad deviations of pore size indicate a non-homogeneous pore structure (Fig. 3b). With a further increase of pressure to 30 MPa, the pore diameter goes down to the submicrometer range and the pores become almost homogeneous. The observed behaviour of pore diameter follows the prediction of classical nucleation theory for homogeneous nucleation^48^. For pressures above 30 MPa the diameter of the pores does not further decrease, as the energy barrier for nucleation, critical nucleus radius and nucleation rate reach a plateau around their minimum^37^. Thus, neither smaller pores nor higher cell density are obtained, but increasing the saturation pressure to 50 MPa allows for shorter saturation times of 2 hours.

### Optimising Pore Morphology through Saturation Temperature

**Figure 4:**
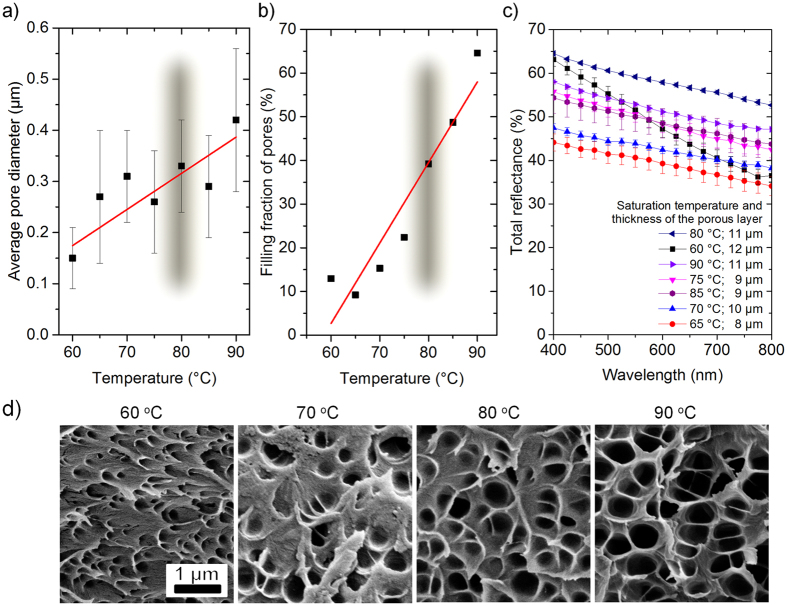
Optimising pore morphology in terms of reflectance through saturation temperature at a fixed pressure of 50 MPa. Dependence of (**a**) pore diameter, (**b**) pore fraction and (**c**) total reflectance on saturation temperature. The temperature range, resulting in the best whiteness, is shadowed (in (**a**) and (**b)**). The thickness of the scattering porous layers is also provided (in (**c**)). The whitest films are obtained at 80 °C. (**d**) SEM images of the porous films, showing that pore size and filling fraction grow with temperature (shown above each SEM image).

After optimising the saturation pressure, we improved the process temperature to obtain the whitest porous films. Thin transparent PMMA films with a nominal thickness of 7 *μ*m were spincoated on a glass slide and saturated at 50 MPa for 2 h at variable temperature. With increasing saturation temperature, Δ*T* is also rising, reducing vitrification pressure and, thus, increasing the growth’s time for the pores, their size and filling fraction^37^. So, within the tested temperature range (from 60 °C to 90 °C) the pore diameter increases by 2.8 times (Fig. 4a and d) and the pore fraction grows by 6 times (Fig. 4b). Therefore, a quite broad distribution of the morphological features can be obtained by varying the temperature.

The total reflectance of the films with different morphologies was measured in the visible range between 400 nm and 800 nm and analysed with respect to the thickness of the porous scattering layer (Fig. 4c). The best total reflectance of about 60% at 600 nm, which is a mean value of the applied measurement range, was obtained for the 11 *μ*m layer at 80 °C, corresponding to a pore diameter of 339 ± 109 nm (Fig. 4a) and the a filling fraction of 39% (Fig. 4b). Although the layer obtained at 60 °C shows high reflectance at 400 nm, for longer wavelengths it demonstrates a strong decay of about 40%, suggesting that small pores and low filling fractions are less efficient for broadband light scattering.

### Optical properties of the porous films

We also analysed the thickness dependent optical properties of the porous films. Following the above described procedure (Fig. 2a), we spin-coated PMMA films of different nominal thicknesses between 7 *μ*m and 50 *μ*m on glass substrates and saturated them with 50 MPa SC-CO_2_ pressure at 80 °C for 2 h. As one can expect, the thickness of the resulting porous film is always larger than the initial one. All films have dense pores in the centre with a transition to a pore-free layer on the top surface (see the inset in Fig. 2b) caused by a reduced concentration of CO_2_ molecules close to the open tip surface during depressurisation. Therefore, the thickness of this pore-free layer at the top open surface depends on the speed of the CO_2_ depressurisation. The slower that process the thicker the pore-free layer. The bottom surface on the other hand does not have a pore-free layer due to the close contact provided by the spin-coating process. The thickness of the pore-free layer at the top surface, when all other process conditions are fixed, depends on the sealing between PMMA film and the covering glass (Fig. 2a), which varies due to surface micro roughness and is nearly independent of the sample’s thickness. The thickness of the pore-free layer is not homogeneous and varies between 1 *μ*m to 2 *μ*m. In nature, similar pore-free layers also surround the scattering scales of white beetles^10^ which most likely serve for mechanical stability as well as the protection of the porous structure. As solid PMMA does not significantly absorb light over the visible spectrum, the pore-free layer should not significantly contribute to reflectance and, therefore, was subtracted from the film thickness in the following analysis. The thicknesses of the porous scattering layers were between 9 *μ*m and 79 *μ*m.

**Figure 5:**
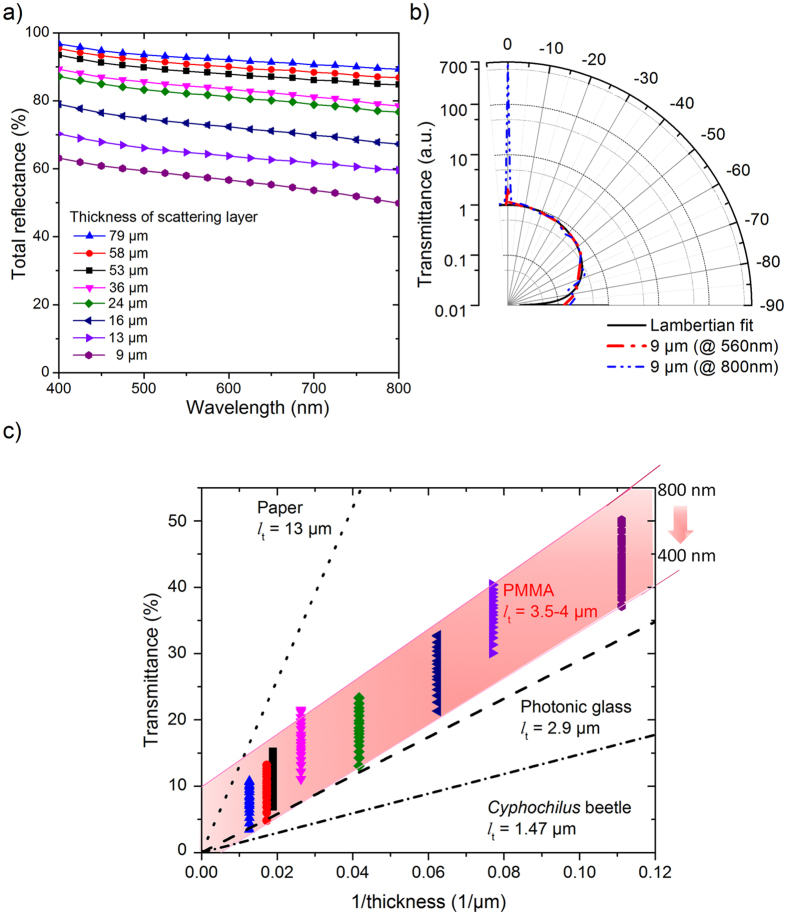
(**a**) Total reflectance of foamed PMMA films with various thicknesses as a function of wavelength. The nearly constant reflectance for all wavelengths leads to the bright white optical appearance of the films. For a thickness of 16 *μ*m of the scattering layer the total reflectance is about 70% corresponding to the value reported for the white *Cyphochilus* beetle (see Fig. S3 in ref. 10). Even a layer thickness of 9 *μ*m reflects about 57% at 600 nm. (**b**) Angular distribution of transmitted light with wavelengths of 560 nm and 800 nm for a porous 9 *μ*m thin scattering layer. The diffused part of the transmittance is normalised and fitted with a Lambertian scattering profile. (**c**) Plot of the transmittance vs. the inverse film thickness using the data shown in (**a**). This representation is also known as “Ohm’s law for light” and allows a comparison of scattering mean free path (*l*_t_) of the foamed PMMA films with prototypes of white materials like paper, photonic glass and the *Cyphochilus* beetle. (Values of *l*_t_ for paper, photonic glass and *Cyphochilus* beetle are taken from ref. 25) The *l*_t_ of the porous PMMA is wavelength-dependent, being around 4 *μ*m at 800 nm and 3.5 *μ*m at 800 nm (shadowed in **c**).

Measuring the total reflectance in the visible range from 400 nm to 800 nm, we observed no significant peaks and a nearly constant total reflectance. As it can be expected, the total reflectance increases with the sample thickness (Fig. 5a) from 57% to 90% at 600 nm for the scattering layers with thicknesses of 9 ± 1 *μ*m and 53 ± 2 *μ*m, respectively. There is a decay in reflectance for longer wavelengths, which is more pronounced for thinner films. The reflectance at 800 nm is 13% and 7% lower than at 400 nm for the 9 *μ*m and 53 *μ*m thick porous layers, respectively. Nonetheless, it is interesting to note that these thin porous layers already effect such an efficient white reflectance.

In order to get insight into the far-field profile of the scattered light, we measured the angular distribution of the transmitted light for 560 nm and 800 nm for a 9 *μ*m thick porous PMMA layer (Fig. 5b). The diffused component of the transmitted light was normalised and fitted with a Lambertian profile. A non-negligible fraction of the light is specularly transmitted leading to the high transmittance at 0° for both wavelength. The ratio between the specular and diffuse components of the transmittance increases with wavelength (×2.4 for 560 nm and ×673 for 800 nm). The reason for this effect is most likely the non-optimal pore morphology for higher wavelengths. With increasing angle, the transmitted light intensity gradually decreases. The measured scattered light profile follows closely that of a Lambertian surface in air. Consequently, the thin white PMMA films might serve as light management elements like potential light diffusers either at the backside of thin film solar cells, to elongate the optical path length^26^, or in LEDs for outcoupling the generated light and modifying its emission pattern^33^.

We compared the overall whiteness of the presented porous films with other prototypes of white materials, by their effective transport mean free path *l*_t_, i.e. the average distance within the sample after which light gets scattered. The smaller the *l*_t_ the more efficient is the scattering process within the material. According to Burresi *et al*.^25^, *l*_t_ can be determined as the slope of the curve connecting the total transmittance values, measured for samples of different thickness, plotted vs. inverse sample’s thickness (Fig. 5c). Such representation is also known as “Ohm’s law for light” and allows a comparison with other white materials like paper, photonic glass and the *Cyphochilus* beetle^25^,^24^. Although a use of the “Ohm’s law for light” is less accurate for optically thin films (with thicknesses smaller than 8*l_t_^49^), it is still in use for very thin disordered structures^50^. Here, we assume that all porous layers have similar microstructure, and PMMA has negligible absorption in the visible range, so all not-reflected light is transmitted.

For the measured samples, the transmittance, recalculated from total reflectance, depends on the wavelength of the incoming light, which was between 400 nm and 800 nm. The transmittance data for each sample is shown as vertical lines, covering the mentioned wavelength range. Thus, *l*_t_ is also wavelength dependent with a value between 3.5 *μ*m to 4 *μ*m for the analysed wavelength range. The thin white PMMA porous layers are close to the best known biological example of whiteness the *Cyphochilus* beetle and nearly as good as photonic glass widely used in optical applications as Lambertian-like reflectors and much better than paper^25^.

### Simulation of scattering in a porous film

**Figure 6:**
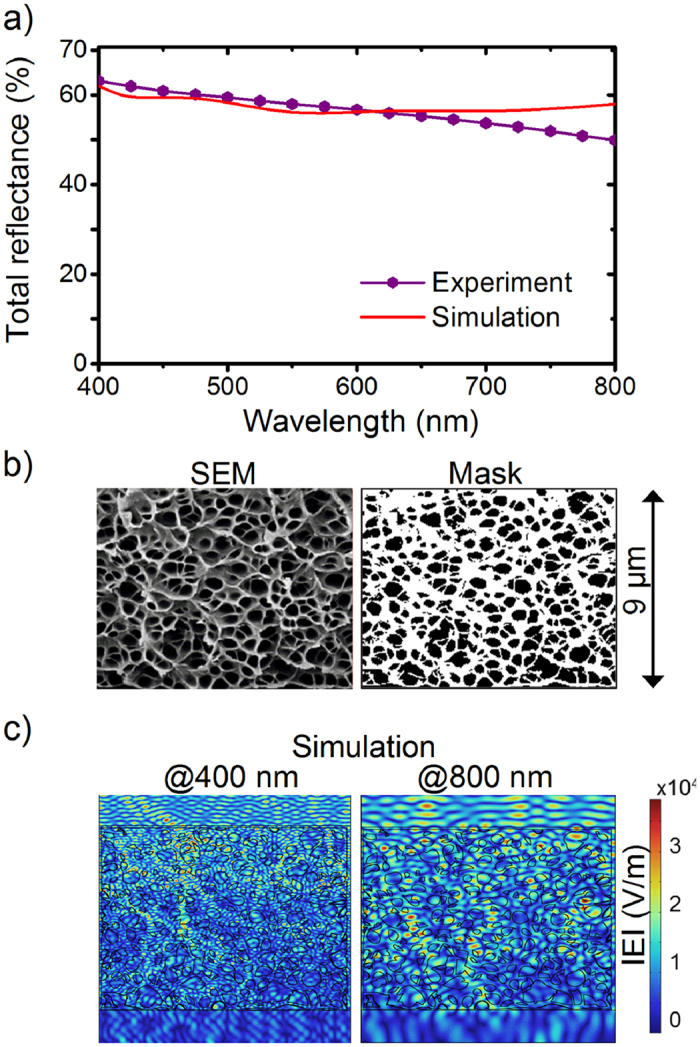
Simulation of the scattering in a porous film. (**a**) Simulated (solid line) and experimental (solid line with dots) total reflectance of a porous 9 *μ*m thick PMMA scattering layer as a function of wavelength calculated with the mask shown in (b). (**b**) An arbitrary chosen area from a SEM cross-sectional image of a 9 *μ*m thick sample and the binary mask of polymer (white) and holes (black) used in the simulations. (**c**) Normalised electric field map of light extracted for short (400 nm) and long (800 nm) wavelengths. Scattering events are dominated at the top section of the film at 400 nm whereas such events are scattered throughout the film at 800 nm demonstrating the influence of the film thickness on wavelength dependent scattering.

In order to get insight into the scattering process of the porous films, we performed 2D optical modelling of the porous layer and calculated the total reflectance (Fig. 6a) in the following way. SEM images of the porous layer were used to determine masks for subsequent finite element method (FEM) simulations (Fig. 6b). The reflectance was calculated for wavelengths in the visible range. The normalised electric field map (Fig. 6c) for 400 nm demonstrates that scattering events dominate within the first half of the sample’s thickness. For light with the double wavelength of 800 nm, however, scattering events spread till the bottom of the film reducing the back scattered part. This outcome is in agreement with Mie scattering theory. In general, Mie scattering is wavelength independent when the size of the scatterer is bigger than wavelength. If this condition is not fulfilled, i.e., the wavelength is comparable to the scatterer, it depends on the wavelength. As our samples have pore diameters between 220 nm and 450 nm, the longer wavelengths of green and red light are less effectively scattered back. Consequently, the total reflectance decreases with wavelength. Aside from small discrepancies above 650 nm, the simulated spectrum is in good agreement with the experimental one, even though a limited simulation mask (9 × 13 *μ*m^2^) was considered. This outcome indicates the overall validity of the applied model.

### Thermomolding of thin porous white films

**Figure 7:**
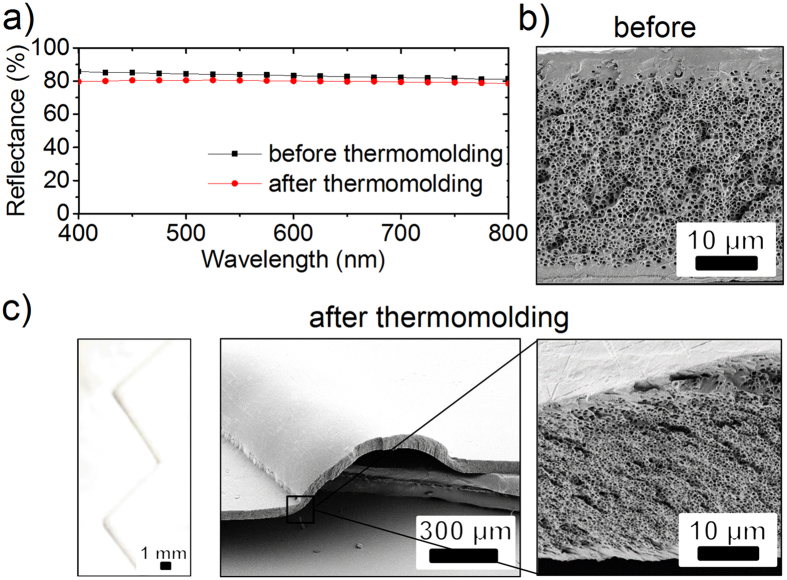
A comparison of white, porous PMMA films before and after thermomolding. (**a**) The total reflectance decreases from 83% to 80% on average. The morphology of pores (**b**) before and (**c**) after thermomolding, observed in side view SEM images, does not change significantly. The thickness of the porous layer, however, decreases slightly. The optical image in (**c**) shows a white microfluidic channel in zigzag form fabricated from a foamed film with a thickness of 159 ± 18 *μ*m.

For practical applications, subsequent structuring of the presented white thin polymeric films is of utmost interest. We applied thermomolding^51^ as one of the most popular methods to shape polymeric films in order to demonstrate that such a treatment does not harm the pores and, therefore, the whiteness of the foils (Fig. 7). For that test, we prepared transparent free standing PMMA films of 57 ± 3 *μ*m thickness by thermal flattening of as-received 500 *μ*m thick PMMA sheets. These thinned films were cut into pieces of 20 × 20 mm^2^ size and saturated in SC-CO_2_ at a pressure of 50 MPa and a temperature of 75 °C for 2 h. These flat and porous white films feature homogeneous nanopores with a diameter of about 250 nm in the porous layer (Fig. 7b). After saturation the thickness of the films increased by a factor of 2.8, to 159 ± 18 *μ*m, due to the formation of the pores. Due to the imperfect sealing at top and bottom, a pore-free skin layer varying between some nanometers to 5 *μ*m forms at both sides of the samples. As there are no scatterers in this pore-free layer, it should not significantly contribute to the total reflectance, considering that PMMA has no significant absorbance in the visible range. At the same time, such a pore-free layer serves for protection of the pores against wear and increases the durability of the structures.

As an example, the films were thermomolded to a microfluidic channel with a zigzag shape in the next step. Within the executed range of the molding parameters (80–90 kN and 100–120 °C for 6 to 10 minutes) all the obtained films stay perfectly white after thermomolding. The best imprint quality was obtained with an applied force of 90 kN at 120 °C applied for 6 minutes. The molded channel looks still perfectly white to the naked eye. The total reflectance is about 80% compared to 83% of the virgin foil (Fig. 7a). Areas highly stressed during the thermomolding process show a slight decrease in film thickness, but no significant polymer flow was observed. Although one might expect deformed pores and increased pore-to-pore distances as a side-product of thermomolding, the resulting films do not show an evident change of pore morphology (Fig. 7c), even when the molding temperature was above the glass temperature for untreated PMMA (*T*_g_ = 105 °C). Here, the molding temperature is measured on the surface of the molding tools, and the temperature inside the polymeric foam itself is unknown (as there is sensor inside the foam). As a rule of thumb, the temperature in the polymer should be about 10 °C less, which would still give a temperature above *T*_g_ of PMMA. At this point we can only speculate what might be the reason. As Wu *et al*. suggested^52^, the glass transition temperature for nanoporous PMMA might increase, due to less effective heat transfer, caused by multiple interphases between the polymer and air trapped in pores. At the same time very thin PMMA films of about 10 nm to 100 nm were reported^53^ to have an decreased/increased *T*_g_ depending on the mold material. Nonetheless, our results show that thin white polymeric films can be further processed with a standard molding technique without significant loss of their whiteness.

## Conclusions

Inspired by natural examples of surfaces appearing white due to scattering, we created ultra-thin white PMMA films. The saturation of PMMA with CO_2_ was optimised to obtain the homogeneous nanoporous films with high reflectance and whiteness. Even 9 *μ*m thin porous layer appear white with a reflectance of 57% and layers with thicknesses over 58 *μ*m feature more than 90% reflectance. The scattering of incoming light on the sub-micrometer pores strongly depends on pore size and is in good agreement with our simulations. Foams with a pore diameter between 220 nm and 450 nm show very good scattering efficiency for lower wavelength. However, such a foam scatters less light for longer wavelength when the pore size is about a half of the light wavelength.

The transport mean free path in the white films is slightly lower than in photonic glass which is the best known artificial scatterer. Nonetheless, the angular dependent reflection of the thin white films is very close to a Lambertian profile, opening interesting applications such as back scattering reflectors for solar cells and light diffusers attached to the front of LEDs. The whiteness of the porous PMMA can be potentially improved by, for example, anisotropic pores^54^ and finer tuning of pressure-temperature conditions. Currently, a 20 *μ*m thick PMMA scattering layer performs as good as 5 *μ*m to 15 *μ*m thick scales of the *Cyphochilus* beetle^25^, with promising options to produce high volumes of material in one production step.

In addition, the white PMMA films might be casted on given substrates or produced as free-standing, flexible foils, which is important for their potential integration on curved surfaces. Still the foaming process can be well controlled and the overall scattering structure should be highly reproducible. The resulting films are naturally covered by a pore-free protective layer, improving their mechanical stability and durability. Finally, foamed films can be further processed by techniques like thermomolding and still remain white and highly refractive. The overall approach of foaming is not limited to the presented case of PMMA, it can be extended to a broad range of thermoplasts^36^.

## Methods

### Preparation of thin PMMA films

As purchased PMMA resist (AR-P 672.11, ALLRESIST GmbH) was spincoated with various thicknesses between 5 *μ*m and 50 *μ*m on a glass slide. All films were transparent at this step (Fig. 2b). In order to avoid thermal deformations and overexpansion the films were covered with the second glass slide and clamped between two neodymium magnets. Such a stack was placed in a home-built high pressure cell^55^ connected to a CO_2_ balloon and a pump, which can compress CO_2_ up to 50 MPa. The polymeric foils were saturated in subcritical and supercritical CO_2_ (critical point at 31 °C and 7.38 MPa) at various conditions, e.g., pressures between 3 and 50 MPa, temperatures between 30 °C to 120 °C and duration times ranging from 2 to 24 hours. Finally, the pores appeared inside the PMMA foil during rapid depressurisation. Depending on the obtained pore morphology, the optical appearance of the foils changed from transparent to milky or deep white.

### Morphological analysis

The thickness of porous films (peeled off from the glass support) was measured at five positions for each sample with Incremental Measuring probe MT60 M (Heidenhain) with a precision of ±0.5 *μ*m and the average value was used. The film thicknesses measured in such a way were in agreement with the values determined from scanning electron microscopy (SEM) images of the film’s cross sections taken with a SUPRA 60 VP (Zeiss). The morphology of the porous films, i.e., pore mean diameter and pore fraction, was also characterised by SEM with a SUPRA 60 VP (Zeiss). Prior to the SEM analysis, the samples were cooled down in liquid nitrogen and cracked. The surface of interest was sputtered with silver for 100 s at 25 mA with a Sputter-Coater (K575X, Emitech) in order to avoid charging effects and ensure a good resolution. The open source software ImageJ^56^ with in-built “Analyse” plugin was applied for automated analysis of pore mean diameter and mean area.

### Optical experimental analysis

The quantitative optical characterization of the foils with different thicknesses was performed with a UV-Vis spectrometer Lambda 1050 (PerkinElmer Inc.). The reflection at the glass/air interface was suppressed by coating the substrate’s rear side with a black absorber. The total (specular + diffuse) reflectance and transmittance spectra were measured in the visible range at close to normal incidence (8° for reflection) with an integrating sphere at three locations for each sample, and the resulting averaged spectra were analysed. A maximum possible spot size of about 10 mm^2^ was used. All measurements were recorded with unpolarised light and referenced with a standard white spectralon.

The angle-resolved transmission profile was measured by using a variable angle spectroscopic ellipsometer (VASE, J. A. Woollam Co., Inc.) running with the software WVASE32 (J. A. Woollam, Ver. 3.774). For that purpose, unpolarised light with a wavelength of 560 nm or 800 nm hits the sample’s frontside at a fixed, normal angle of incidence, and the light, emerging out of the sample’s backside, was collected by a rotating detector between +5° and −90°. In order to only measure the intensity above a threshold value of 10^−6^ and to adjust the number of revolutions per measurements, we worked in the dynamic averaging mode. To further reduce the high-frequency noise, the measured curves were smoothened using a FFT filter available in OriginPro 8. The data, first processed using a FFT filter to reduce the noise, was then normalised so that the profile of the scattered transmitted light can be easily compared with a Lambertian profile used as a benchmark.

### Optical simulations

2D modelling of the bio-inspired porous thin films was performed with the finite element method (FEM) software COMSOL Multiphysics. More details on the modelling can be found in ref. 57. In order to consider a realistic geometry, SEM images of porous thin films were converted to black and white (i.e., binary) images with ImageJ^56^ and subsequently used as a mask for the simulations. The converted mask of around 9 *μ*m × 13 *μ*m was considered in the unit cell and periodic Floquet boundary conditions were applied on the right and left side of the cell. The unit cell was surrounded by 4 *μ*m air medium followed by 400 nm thick perfectly matched layers (PML) on top and bottom, to confine the computational domain. The wavelength dependent optical indices of PMMA were taken from literature^42^. The maximum mesh element size was considered 1/20^th^ of the smallest wavelength of inspection (400 nm).

No optical losses were considered. The incident field was defined as a plane transverse magnetic (TM) wave with unit amplitude and intensity. The normal angle of light incidence was considered for the simulation and therefore, unpolarised light condition was maintained. The total reflectance was calculated by integrating the near-field scattered field (Poynting vector) over the top boundary before PML.

## Additional Information

**How to cite this article:** Syurik, J. *et al*. Bio-inspired, large scale, highly-scattering films for nanoparticle-alternative white surfaces. *Sci. Rep.* **7**, 46637; doi: 10.1038/srep46637 (2017).

**Publisher's note:** Springer Nature remains neutral with regard to jurisdictional claims in published maps and institutional affiliations.

## Figures and Tables

**Figure 1:**
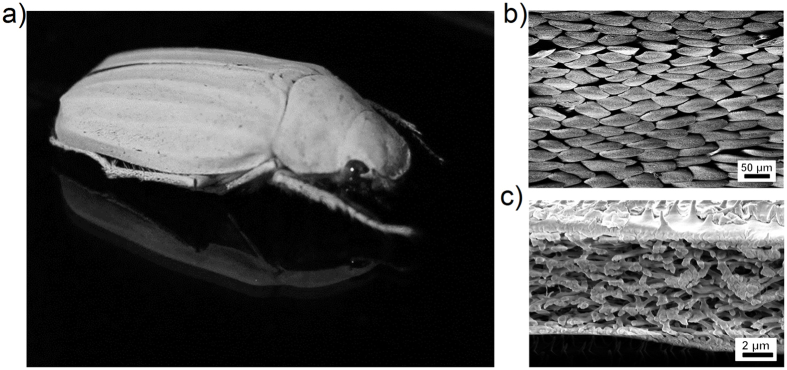
(**a**) The white beetle *Cyphochilus insulanus* is a well-known example for whiteness found in nature. Its body length is about 25 mm–30 mm. (**b**) SEM image of the white scales, covering the legs, head and the body of the *Cyphochilus insulanus* beetle. (**c**) Sectional view of the scale causing the bright white color by multiple light scattering at the random structure of microrods. The upper side of the scales is covered by small spikes while their bottom is almost flat.
